# Accuracy of Longitudinal Assessment of Visceral Adipose Tissue by Dual-Energy X-Ray Absorptiometry in Children with Obesity

**DOI:** 10.1155/2019/2193723

**Published:** 2019-11-03

**Authors:** Katrin A. Dias, Joyce S. Ramos, Matthew P. Wallen, Peter S. W. Davies, Peter A. Cain, Gary M. Leong, Charlotte B. Ingul, Jeff S. Coombes, Shelley E. Keating

**Affiliations:** ^1^School of Human Movement and Nutrition Sciences, The University of Queensland, St Lucia, Brisbane, QLD, Australia; ^2^Child Health Research Centre, The University of Queensland, Brisbane, QLD, Australia; ^3^Heart Care Partners, The Wesley Hospital, Brisbane, QLD, Australia; ^4^Nepean Hospital and Nepean Charles Perkins Center Research Hub, University of Sydney, Kingswood, NSW, Australia; ^5^Department of Circulation and Medical Imaging, Norwegian University of Science and Technology, Trondheim, Norway; ^6^Helse Midt-Norge RHF, Strandvegen 1, Stjordal, Norway

## Abstract

**Background:**

Increased visceral adipose tissue (VAT) is strongly associated with cardiometabolic risk factors. Accurate quantification of VAT is available through magnetic resonance imaging (MRI), which incurs a significant financial and time burden. We aimed to assess the accuracy of dual-energy X-ray absorptiometry- (DXA-) derived VAT (DXA-VAT) against a gold standard MRI protocol (MRI-VAT) in children with normal weight and obesity cross-sectionally, and over the course of a lifestyle intervention.

**Methodology:**

MRI-VAT and DXA-VAT were quantified in 61 children (30 normal weight and 31 with obesity) at baseline. Children with obesity entered a three-month exercise and/or nutrition intervention after which VAT was reassessed. MRI- and DXA-VAT cross-sectional area, volume, and mass were quantified, and associations were calculated at baseline (*n* = 61) and pre-post intervention (*n* = 28, 3 participants dropped out). Method agreement was assessed through Bland–Altman analysis, linear regression, and Passing–Bablok regression.

**Results:**

At baseline, all DXA- and MRI-VAT outcomes were strongly associated (*r* = 0.90, *P* < 0.001). However, there were no significant associations between absolute or relative change in DXA- and MRI-VAT outcomes (*r* = 0.25–0.36, *P* > 0.05). DXA significantly overestimated VAT CSA (cross-sectional area), volume, and mass when compared with MRI (*P* < 0.001) at baseline. Significant proportional bias was observed for all DXA-VAT outcomes at baseline and for relative longitudinal changes in DXA-VAT.

**Conclusions:**

Although DXA-VAT outcomes were strongly associated with MRI-VAT outcomes at baseline, estimates were subject to proportional bias in children with obesity and normal weight. DXA lacks validity for detecting changes in VAT among children with obesity. This trial is registered with NCT01991106.

## 1. Introduction

Childhood obesity remains a priority for public health initiatives worldwide [1]. Although the prevalence of this condition has remained constant over the last ten years, up to 17 percent of children remain affected [1]. Although obesity is defined as the presence of excess adipose tissue [2], it is the distribution of this adipose tissue that holds prognostic information regarding cardiometabolic morbidity and mortality. In particular, visceral adipose tissue (VAT), a type of ectopic adipose tissue depot [3], has been associated with cardiometabolic risk factors, including an abnormal lipid profile, impaired pancreatic beta cell function, insulin resistance, hypertension, and left ventricular hypertrophy [4, 5]. Although exercise and/or diet interventions may lead to reductions in VAT [6], accurate imaging techniques are required to quantify this adipose tissue depot and assess the efficacy of lifestyle programs.

Magnetic resonance imaging (MRI) remains the gold standard for assessment of VAT volume, constructed from the cross-sectional area (CSA) of multiple slices [7]. However, MRI equipment is costly, image acquisition requires a high level of technical expertise and significantly more time than a DXA scan (∼20 minutes versus ∼7 minutes), and scans must be analysed using specialised software by an experienced observer [7]. Dual-energy X-ray absorptiometry (DXA) technology manufactured by Hologic Inc. (Bedford, MA, USA) offers a new software solution, Advanced Body Composition™ with InnerCore™ Visceral Fat Assessment (Version 4.5.3), to estimate VAT. High accuracy for this software was reported when compared cross-sectionally with single-slice computed tomography (CT) derived VAT in a cohort of overweight adult women [8] and single-slice MRI-derived VAT in 2,689 adults [9]. Importantly, DXA is less expensive and more readily available than MRI. As such, accurate quantification of VAT using DXA Advanced Body Composition™ with InnerCore™ Visceral Fat Assessment would increase the feasibility of assessing VAT as a clinical outcome, in both research and clinical settings. However, it is paramount that VAT assessment using DXA is accurate and is valid in its ability to track longitudinal changes resulting from interventions that target obesity-related cardiovascular disease risk [9]. To our knowledge, no published studies have investigated the accuracy of InnerCore™ Visceral Fat Assessment in children. Furthermore, there is currently no evidence regarding the accuracy of longitudinal changes in VAT measured by DXA. Therefore, we aimed to determine the relationship between (1) VAT derived from MRI (MRI-VAT) and DXA (DXA-VAT) in children with normal weight and obesity and (2) longitudinal changes in VAT derived from MRI and DXA in children with obesity.

## 2. Materials and Methods

Thirty-one children with obesity (BMI ≥ percentile curves that pass through 30 kg/m^2^ at the age of 18 years, International Obesity Task Force criteria) [10] and thirty normal-weight children (BMI percentile curves that pass through 18–25 kg/m^2^ at the age of 18 years, International Obesity Task Force criteria) [10] aged 7–16 years were recruited as part of an international multicentre randomized controlled trial at the University of Queensland, Brisbane, Australia. The study was approved by the University of Queensland Human Research Ethics Committee (reference number 2013000539) and the Mater Hospital Human Research Ethics Committee (reference number HREC/13/MHS/119/AM01). Participants' legal guardians approved consent, and participants provided written assent prior to participation. Inclusion and exclusion criteria have been previously described [11]. In brief, research staff completed anthropometric measurements including height, weight, waist, and hip circumference. Pubertal status was self-reported through visual identification of Tanner stages of puberty which has been validated against nurse evaluations [12, 13]. Baseline assessments were completed in all participants (*n* = 61). Children with obesity were randomized and completed a three-month exercise and/or nutrition intervention after which they were reassessed (*n* = 28). Details of the intervention protocol have been previously published [11]. Three participants dropped out of the study and did not complete postintervention assessments (exercise and nutrition, *n* = 1; nutrition only, *n* = 2). The baseline characteristics of participants who dropped out of the trial were not significantly different from trial completers.

### 2.1. Dual-Energy X-Ray Absorptiometry

**Figure 1:**
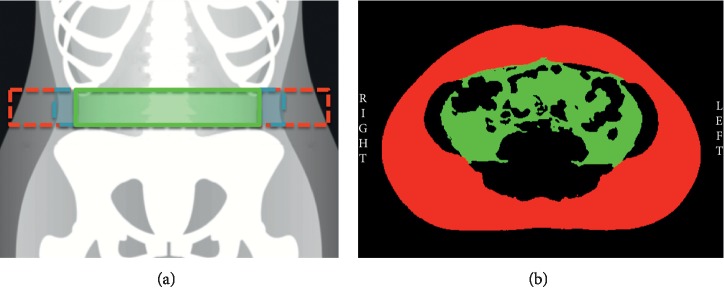
Subcutaneous (red) and visceral (green) adipose tissue compartments shown on DXA (a) and MRI (b). Blue dashed lines on DXA panel show the abdominal wall musculature.

Whole body composition was assessed using a Discovery DXA System, QDR Series (Hologic Inc., Bedford, MA, USA). Body fat percentage, and absolute and relative android fat were quantified using APEX software (Version 4.5.3, Hologic Inc., Bedford, MA, USA), whereas VAT outcomes were estimated using InnerCore™ Visceral Fat Assessment from a 5.2 cm wide section (superior to inferior), extending upwards from the top of the iliac crest in all participants, regardless of age or sex (Version 4.5.3, Hologic Inc., Bedford, MA, USA). The software estimates VAT mass, volume, and cross-sectional area using X-ray derived pixel positions from a two-dimensional sagittal image of a body slice containing visceral and subcutaneous fat (Figure 1). Measurements were postprocessed with manual editing when appropriate as per manufacturer guidelines [14]. All scans were conducted and analysed by a single trained investigator. The technical error of DXA scans is ∼1%.

### 2.2. Magnetic Resonance Imaging

Axial images were acquired using a 1.5T MRI system (Siemens Symphony Sonata, Siemens, Erlangen, Germany) equipped with a six-channel body matrix coil and a six-channel spine coil by a technician blinded to group allocation. Subjects were positioned supine inside the bore, and images were acquired using true fast imaging steady-state precision technique with breath hold (repetition time = 3.76 ms; echo time = 1.88 ms; flip angle 75°; matrix = 220 × 256; rectangular field of view (FOV) = 400 mm × 400 mm; slice thickness = 8 mm; 14 slices; acquisition time = 12 s). Fourteen, 8mm thick axial slices centred over the umbilicus were acquired during breath hold. MRI scans were anonymised and analysed by a single investigator using SliceOmatic (version 5.0; Tomovision, Magog, Canada). To closely match the region analysed by DXA for VAT, MRI localiser scans were used to select two slices centred over L4/L5 (umbilicus) and average VAT area was calculated (Figure 1). MRI-VAT volume was quantified from 6 slices centred over L4/L5 to closely approximate the measurement site of DXA-VAT outcomes. MRI-VAT mass was calculated as the product of MRI-VAT volume and the density of adipose tissue (0.90 g/cc). Although single-slice CSA is most commonly reported in the literature, multi-slice volume and mass are considered the gold standard because of reduced variability and increased clinical relevance [7]. Therefore, all three VAT outcomes were analysed and reported. Nine MRI scans were randomly selected and reanalysed to calculate the intra-observer coefficient of variation for VAT (3.6%).

### 2.3. Statistical Analysis

Data are expressed as mean ± SD if continuous and normally distributed or median (IQR) and percentages if categorical and nonnormally distributed. SPSS Statistics (version 24.0, IBM, NY, USA) and MedCalc (version 16.8.4, MedCalc Software, Ostend, Belgium) were used to perform all statistical analyses. For participants who completed the intervention (children with obesity, *n* = 28), a dependent sample *t*-test was used to examine within-group changes in VAT. Univariate regression modelling was used to determine the association between MRI- and DXA-VAT outcomes in all participants at baseline (*n* = 61), where MRI-VAT outcomes were entered as independent variables and DXA-VAT outcomes were entered as dependent variables. Identical analyses quantified the relationship between MRI- and DXA-quantified absolute (post-VAT − pre-VAT) and relative (post-VAT − pre-VAT/pre-VAT) change in VAT outcomes in children with obesity (*n* = 28). Baseline, change in absolute, and change in relative percent body fat were entered as independent predictor variables into the respective multiple regression models due to the strong relationship between percent body fat and MRI-VAT outcomes (*r* = 0.80–0.82). Univariate regression analyses were repeated separately for each tertile of (1) percent body fat, (2) absolute, and (3) relative change in percent body fat. Bland–Altman analysis was used to assess the level of agreement of VAT outcomes between the two instruments at baseline and for change data. If data appeared nonnormally distributed following visual inspection of the Bland–Altman plot, a Passing–Bablok regression was used to assess the level of agreement between the methodologies. The Passing–Bablok regression is preferential to the Deming regression as it does not assume that measurement error is normally distributed and is robust against outliers [15]. For both Bland–Altman and Passing–Bablok regression analyses, systematic and proportional bias was assessed. Proportional bias refers to the bias that is related to the magnitude of the value measured, as defined by Ludbrook (1997) [16]. Using the Bland–Altman plot, systematic bias was determined if the line of equality (*y* = 0) was not in the 95% CI of the mean difference while proportional bias was assessed by visually inspecting the regression line of differences. Using the Passing–Bablok regression, systematic bias was determined if the 95% CI of the intercept did not contain the value 0. Proportional bias of the new method (DXA) from the reference method (MRI) was assessed using linear regression analysis for normally distributed data, or Passing–Bablok regression analysis for nonnormally distributed data. Using the Passing–Bablok regression, proportional bias was determined if the 95% CI of the intercept did not contain the value 1. The strength of the correlation coefficients was determined as small (*r* ≤ 0.1), medium (*r* = 0.3–0.5), or large (*r* > 0.5) [17]. All statistical tests with *P* value < 0.05 were considered statistically significant.

## 3. Results

### 3.1. Patient Characteristics

Table 1 summarises baseline clinical and anthropometric characteristics in all children (*n* = 61), children with obesity (*n* = 31), and normal-weight children (*n* = 30) at baseline, and change data for children with obesity pre-post intervention (*n* = 28).

### 3.2. Accuracy of Cross-Sectional DXA-VAT Assessment

**Figure 2:**
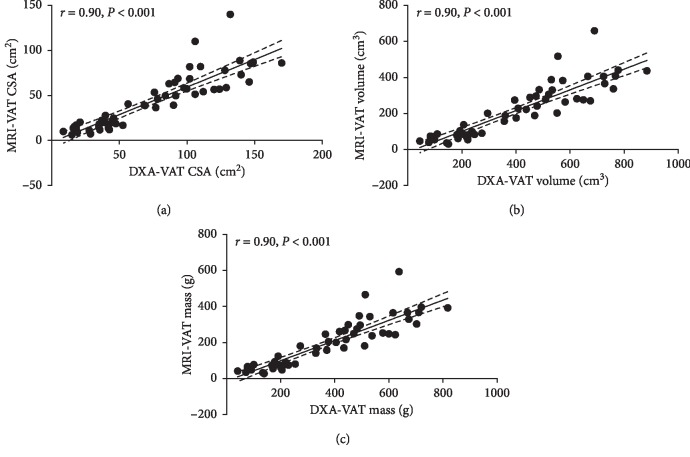
Linear regression illustrating the relationship between MRI- and DXA-VAT CSA (a), volume (b), and mass (c) in all participants. Dashed lines represent 95% confidence intervals.

DXA-estimated VAT CSA was strongly associated with MRI-quantified VAT CSA (*r* = 0.90, *P* < 0.001) (Table 2; Figure 2(a)). Strong associations were also noted between DXA and MRI for VAT volume and mass (*r* = 0.90, *P* < 0.001) (Table 2; Figures 2(b) and 2(c)). The aforementioned relationships between DXA- and MRI-VAT outcomes remained significant even when percent body fat was accounted for (*P* < 0.001). DXA-VAT CSA and percent body fat (Table 2, Model 2) were able to account for 83% of the variation in MRI-VAT CSA. Similarly, DXA-VAT volume and mass, alongside percent body fat, accounted for 85% of variation in corresponding MRI-VAT outcomes (Table 2, Models 4 and 6). The relationship between DXA- and MRI-VAT outcomes was weakest in the lowest (21.9% ± 4.5% body fat; *r* = 0.50–0.51, *P* < 0.05) and highest (50.0% ± 3.0% body fat; *r* = 0.38–0.47, *P* > 0.05 for CSA, *P* < 0.05 for volume and mass) body fat percent tertiles. The middle tertile (38.9% ± 5.5% body fat; *r* = 0.94, *P* < 0.05) showed the strongest associations between DXA- and MRI-VAT outcomes.

**Figure 3:**
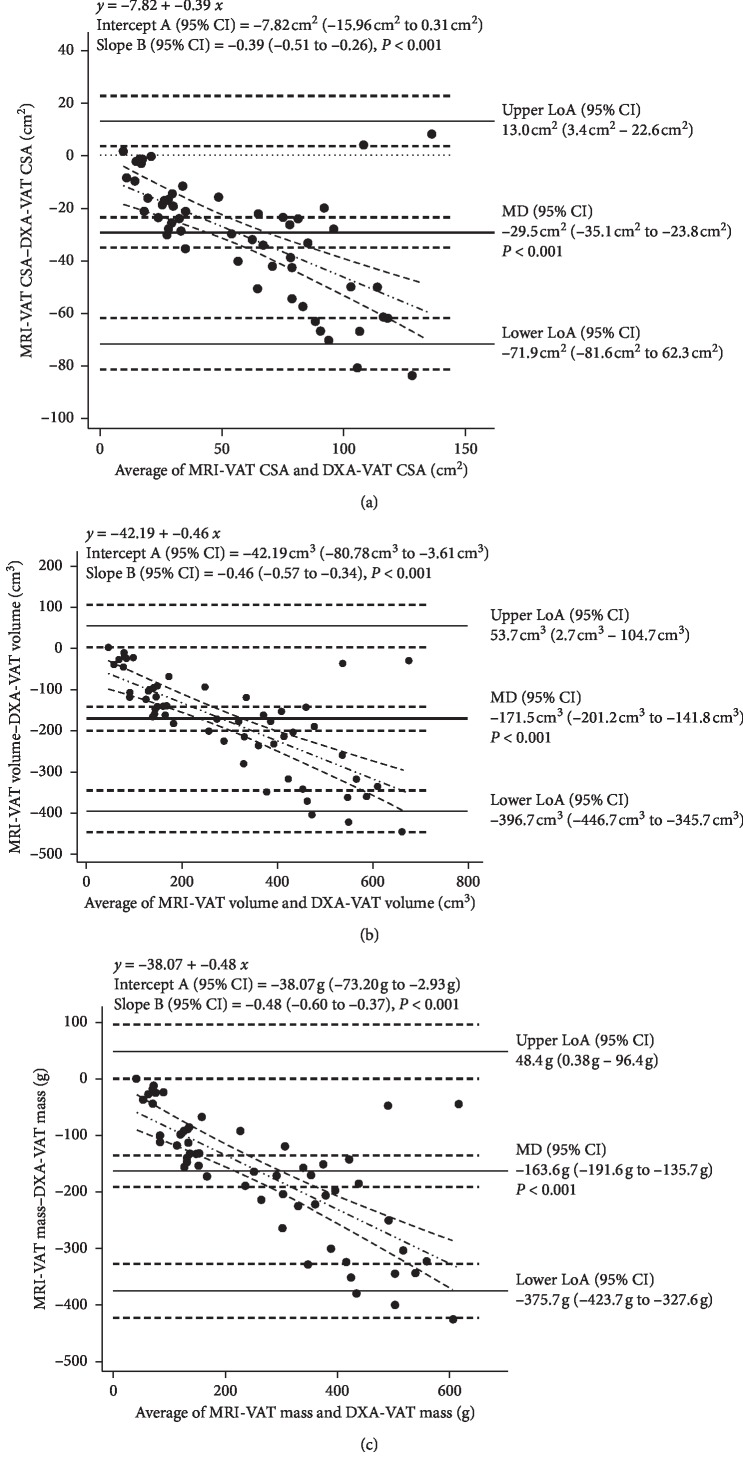
Bland–Altman plots comparing MRI-VAT (reference) with DXA-VAT CSA (a), volume (b), and mass (c) in all participants. The plots show the mean difference between the two measures (thick solid line), the upper and lower limits of agreement (thin solid line), 95% confidence intervals (dashed lines), line of equality (*y* = 0, dotted line), and the regression line of differences (dashed-dotted line). MD = mean difference; LoA = limits of agreement.

**Figure 4:**
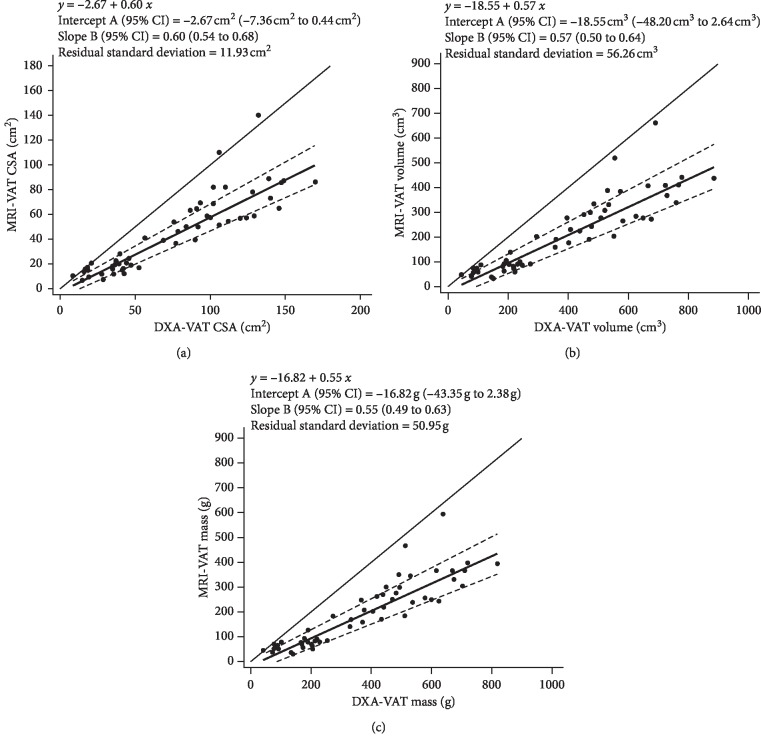
Passing–Bablok regression plots comparing MRI-VAT (reference) with DXA-VAT CSA (a), volume (b), and mass (c) in all participants. The plots show the regression line (solid line), 95% confidence intervals (dashed lines), and identity line (*x* = *y* dotted line).

Bland–Altman plots assessed the level of agreement between DXA- and MRI-VAT outcomes. DXA significantly overestimated VAT when compared with MRI for CSA (29.5 cm^2^ [23.8cm^2^–35.1 cm^2^], *P* < 0.001), volume (171.5 cm^3^ [141.8cm^3^–201.2 cm^3^], *P* < 0.001), and mass (163.6 g [135.7 g–191.6 g], *P* < 0.001) (Figure 3). Because of the heteroscedasticity observed in the Bland–Altman plots, a Passing–Bablok regression was used to assess systematic and proportional bias between methodologies. Whereas systematic bias was no longer present, each DXA-VAT outcome variable illustrated significant proportional bias (Figure 4).

### 3.3. Accuracy of DXA-VAT Assessment to Track Longitudinal Change

**Figure 5:**
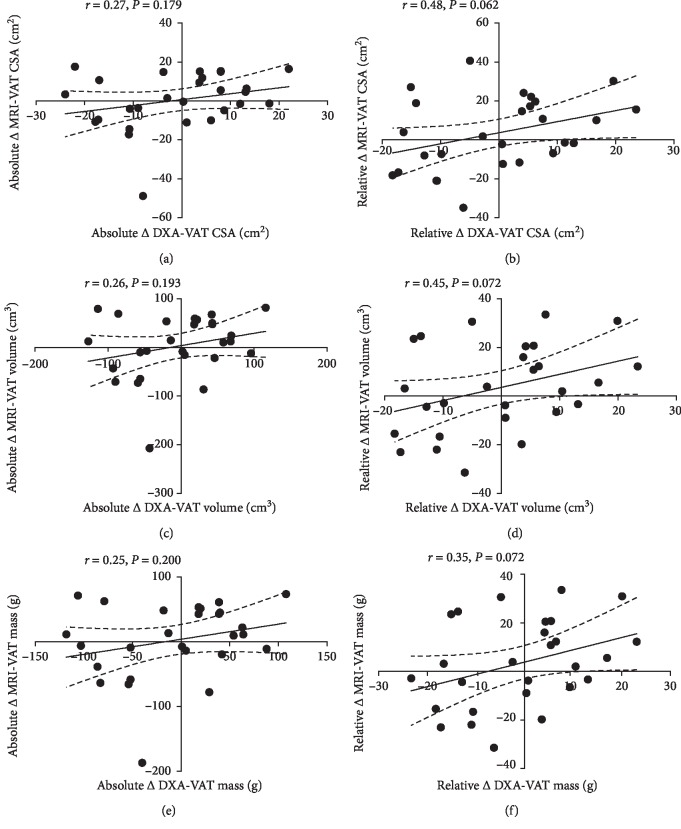
Linear regression illustrating the relationship between absolute and relative change in MRI- and change DXA-CSA (a, b), volume (c, d), and mass (e, f). Dotted lines represent 95% confidence intervals.

There were no significant associations for absolute change in VAT CSA, volume, or mass between DXA and MRI (Table 3, Models 1, 5, and 9, Figures 5(a), 5(c) and 5(e)). This held true when absolute change in percent body fat was entered in the multiple regression (Table 3, Models 2, 6, and 10). The relationship between relative change in MRI-VAT and DXA-VAT for CSA, volume and mass was also nonsignificant (Table 3, Models 3, 7, and 11; Figures 5(b), 5(d) and 5(f)). However, when accounting for relative change in percent body fat alongside relative change in DXA-VAT CSA (Table 3, Model 4), a significant proportion of the variance in MRI-VAT CSA could be explained (*R*^2^ = 0.23, *P*=0.040). Linear regression analysis by tertile of percent body fat illustrated similar findings (results not shown).

**Figure 6:**
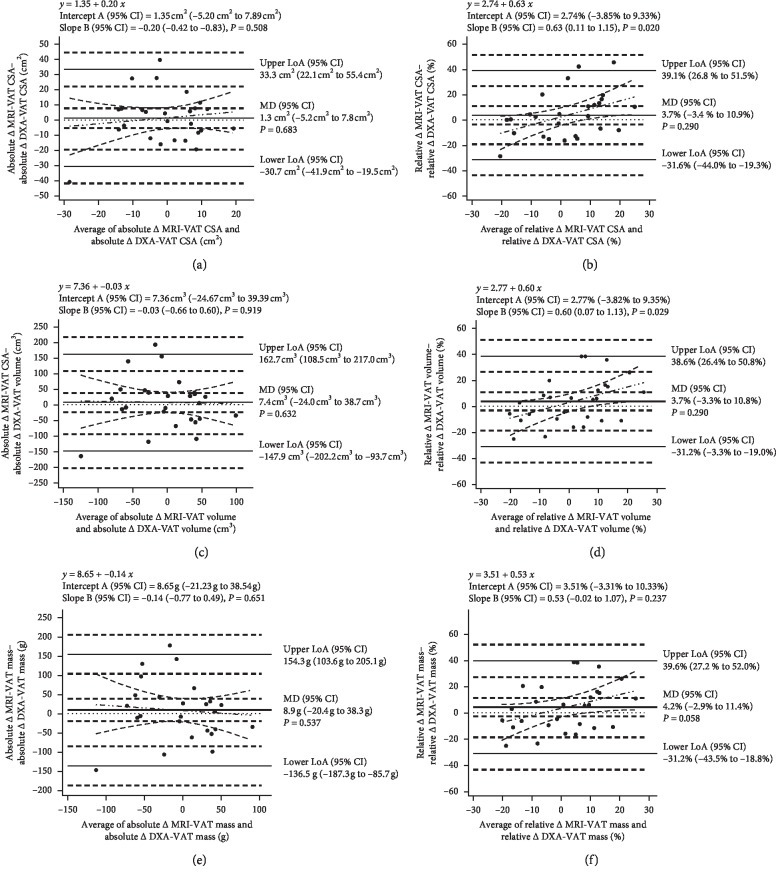
Bland–Altman plots comparing absolute and relative change in MRI-VAT (reference) with absolute and relative change in DXA-VAT CSA (a, b), volume (c, d), and mass (e, f). The plots show the mean difference between the two measures (thick solid line), the upper and lower limits of agreement (thin solid line), 95% confidence intervals (dashed lines), line of equality (*y* = 0, dotted line), and the regression line of differences (dashed dotted line). MD = mean difference; LoA = limits of agreement.

Bland–Altman analysis revealed nonsignificant mean differences between MRI and DXA measurements of absolute and relative change in VAT outcomes (Figure 6). Proportional bias was present for relative change in DXA-VAT CSA and volume compared with MRI-VAT (*P*=0.020 and *P*=0.029, respectively). The remaining change outcomes did not display proportional bias.

## 4. Discussion

This is the first study in children to assess the accuracy of cross-sectional and longitudinal change in DXA-VAT outcomes compared with the gold standard technique, MRI. We observed that DXA-VAT was strongly associated with MRI-VAT. However, DXA displayed significant proportional bias for measurement of VAT CSA, volume, and mass compared with MRI. Our study is the first to investigate and illustrate no association between longitudinal changes in DXA- and MRI-VAT outcomes and the presence of significant proportional bias between relative changes in DXA- and MRI-VAT measurements.

To our knowledge, only two other studies have compared the accuracy of VAT volume derived from DXA with MRI [9, 18], and this is the first study to do so in a pediatric population. Our cross-sectional findings are consistent with previous work examining the correlation between CT and DXA (Hologic) estimated VAT in adults [8, 19]. The association between DXA and MRI-VAT CSA in the present study (*r* = 0.90, *P* < 0.001) is comparable to that of previous findings in 272 [8] and 135 [19] adult women (*r* = 0.93 and *r* = 0.86, *P* < 0.001, respectively). These findings were replicated in a study of 102 older men (61.6 ± 6.5 years) that compared VAT derived from DXA (Lunar Prodigy, GE Healthcare, Madison, WI, USA) with CT and MRI-VAT. The authors found a significant relationship between DXA-VAT volume and CT-VAT area (*r* = 0.83, *P* < 0.001), and DXA- and MRI-VAT volume calculated from 3 slices (*r* = 0.90, *P* < 0.001) [18]. Similarly, we observed a strong relationship between DXA-VAT volume and MRI-VAT volume (*r* = 0.90, *P* < 0.001). Furthermore, using a heterogeneous study cohort, Neeland et al. reported strong relationships between DXA (Hologic) and MRI-VAT mass (*R*^2^ = 0.82–0.86) [9]. This is consistent with the relationship we report between DXA and MRI-VAT mass (*R*^2^ = 0.81, *P* < 0.001). Overall, these data support the utility of DXA to accurately estimate VAT cross-sectionally.

Assessment of agreement between baseline DXA- and MRI-VAT outcome variables revealed significant proportional bias between the instruments. DXA-VAT CSA, volume, and mass significantly overestimated the corresponding MRI measurements by 29.5 cm^2^, 171.5 cm^3^, and 163.6 g, respectively. This bias was proportional where the difference between MRI-VAT and DXA-VAT estimations increased with higher volumes of VAT. Likewise, Cheung et al. reported the presence of approximately 30% proportional bias between MRI-VAT and DXA-VAT in older men [18]. However, in contrast to our finding that DXA overestimates VAT compared to MRI, Cheung et al. (2016) reported that DXA underestimated MRI-VAT volume by 1285 cm^3^ [18]. This discrepancy was likely due to differences in MRI volume measurement. We specifically compared the MRI-VAT volume that most closely reflected the VAT volume estimated by DXA. In our study, MRI-VAT volume was quantified from 6 slices centred over L4/L5 covering a 4.8 cm wide (superior to inferior) section to replicate DXA-VAT volume, which is quantified from a 5.2 wide section. On the contrary, Cheung et al. calculated MRI-VAT volume from a 16 cm window, whereas the Lunar DXA-VAT volume is calculated from a 10 cm window [18]. Using the hologic system, Neeland et al. reported that DXA-VAT modestly underestimated MRI-VAT mass at lower VAT levels and overestimated MRI-VAT at higher VAT levels [9], which was in partial agreement with our findings which showed a weak relationship between DXA- and MRI-VAT in the lowest and highest body fat tertiles. The inconsistency between studies with regard to the presence, magnitude, and direction of bias requires DXA-VAT to be used carefully and interpreted with caution until further clarification is available.

Our study is the first to report the association between longitudinal change in DXA- and MRI-VAT outcomes following a recent request for this type of assessment [9]. Quantification of VAT volume, rather than area, is critical to minimise the error associated with soft tissue movement and increase reliability [7]. Importantly, there was no relationship between MRI and DXA-quantified absolute and relative longitudinal changes in VAT outcomes including volume. Although DXA-VAT outcomes were unaffected by systematic bias, DXA illustrated significant proportional bias for relative changes in VAT CSA and volume. Therefore, until further evidence is available from additional method-comparison studies in larger, more diverse study cohorts, we suggest that DXA not be used as the primary outcome measure for VAT quantification in longitudinal/intervention studies in obese pediatric populations.

Our assessment of the two methodologies was completed in a heterogeneous population with distinct body compositions. Therefore, percent body fat was considered a covariate in linear regression analysis, given the significant relationship between baseline percent body fat and VAT as well as the significant decrease in percent body fat following the lifestyle intervention in children with obesity. In the combined population at baseline, inclusion of percent body in the multiple regression model improved the predictive ability of the DXA-VAT models. Accounting for change in percent body fat in the absolute and relative change models resulted in a significant relationship between relative change in DXA- and MRI-VAT CSA, although the model still only explained 23% of the variance in relative change in MRI-VAT CSA.

A major limitation of the present study is that we did not see any change in MRI-quantified VAT following the intervention, which limits the generalizability of our findings. Furthermore, inherent differences in the MRI and DXA-VAT quantification techniques should be noted. MRI-VAT is calculated from axial slices that display contrasting pixel intensities for adipose and lean tissue. Using semi-automated software, pixels are assigned to VAT based on intensity. The Hologic DXA software, InnerCore™ Visceral Fat Assessment, estimates VAT using X-ray derived pixel positions from a two-dimension sagittal image of a body slice [14]. Therefore, a portion of the bias that we have reported could be attributed to distinct differences in methodologies. Moreover, the Hologic DXA software was developed and validated against single-slice CT even though single-slice imaging has shown to be inaccurate for detecting longitudinal VAT changes [20]. It is therefore likely that this DXA technology, based on an inaccurate measurement for detecting longitudinal change, may not be robust enough to estimate changes in VAT. Moreover, the body composition of children differs from adults, which may have compounded the variability in VAT changes between imaging modalities. We reported a 3.6% coefficient of variation (CV) for MRI-VAT and a recent study reported a 5.1% CV for DXA-VAT [21]. Importantly, the moderate CV for both modalities may have masked any longitudinal changes in VAT in our study cohort. In addition, we may have been underpowered to detect longitudinal changes in VAT due to a small sample size for this particular analysis.

InnerCore™ Visceral Fat Assessment has not been validated in a pediatric population and is not cleared for medical use by the Food and Drug Administration or Therapeutic Goods Association. As such, this investigation is a vital first step in establishing the accuracy of DXA-quantified VAT as this modality has the potential to be used extensively in clinical practice and research. Further studies are warranted to investigate whether similar findings are observed following a more intensive intervention associated with greater longitudinal changes in abdominal adipose tissue, as well as in adult populations. If future studies show significant discrepancies between DXA and MRI derived VAT, a correction factor could be devised to account for the variation between methodologies.

## 5. Conclusions

In conclusion, we found a strong relationship between the cross-sectional assessment of DXA- and MRI-VAT; however, significant proportional bias was present in children with normal weight and obesity. Furthermore, DXA-VAT was unable to accurately track longitudinal changes in MRI-VAT in children with obesity. Therefore, until further data become available, we advise that DXA estimated VAT, using currently available software, not be used for quantifying VAT changes in children with obesity.

## Figures and Tables

**Table 1:** Baseline clinical and anthropometric characteristics in (1) all participants, (2) participants with obesity, (3) healthy-weight participants, and (4) longitudinal change in anthropometric characteristics in participants with obesity.

	All (*n* = 61)	Healthy-weight (*n* = 30)	Obese (*n* = 31)	Δ pre-post intervention (obese, *n* = 28)
Age	11.6 ± 2.0	11.8 ± 2.2	11.4 ± 1.9	
Sex (F, %)	44.3	46.7	41.9	
Tanner puberty stage (1–4)	2 (1.5–3)	2 (1–4)	2 (2‐3)	
Height (cm)	154.7 ± 13.5	154.0 ± 14.9	155.3 ± 12.2	1.9 ± 1.1^*∗∗*^
Height *z*-score	0.94 ± 0.94	0.64 ± 1.00	1.23 ± 0.79	0.00 ± 0.12
Weight (kg)	58.4 ± 20.9	43.0 ± 10.8	73.3 ± 17.2	1.6 ± 3.0^*∗*^
Weight *z*-score	1.66 (0.30–2.55)	0.23 ± 0.58	2.49 ± 0.47	−0.04 ± 1.22
BMI (kg/m^2^)	24.2 (17.5–30.0)	17.8 ± 1.9	30.1 ± 4.3	0.0 ± 1.2
Body fat (%)	39.7 (25.8–46.9)	26.1 ± 7.3	47.7 ± 4.3	−1.1 ± 1.5^*∗∗*^
WC (cm)	76.2 (64.7–89.5)	64.1 ± 5.7	88.6 ± 2.5	−0.3 ± 2.4
WHR	0.82 ± 0.05	0.79 ± 0.04	0.85 ± 0.04	0.00 ± 0.03
WHtR	0.49 (0.41–0.57)	0.42 ± 0.03	0.57 ± 0.05	0.00 ± 0.02
Android fat (kg)	1.2 (0.5–2.7)	0.6 ± 0.3	2.8 ± 0.8	0.0 ± 0.2
Android fat (%)	39.3 (21.8–50.7)	23.3 ± 7.1	49.6 ± 4.3	−1.2 ± 2.2^*∗∗*^
MRI-VAT CSA (cm^2^)	39.0 (15.9–62.0)	17.4 ± 7.7	65.2 ± 23.5	−0.1 ± 14.2
MRI-VAT volume (cm^3^)	181.5 (76.9–295.6)	80.5 ± 34.1	314.8 ± 112.1	0.1 ± 64.6
MRI-VAT mass (g)	163.3 (69.3–266.0)	72.5 ± 30.7	238.2 ± 100.9	0.1 ± 58.2
DXA-VAT CSA (cm^2^)	62.5 (35.8–105.0)	34.0 ± 14.6	107.9 ± 28.3	−0.9 ± 12.6
DXA-VAT volume (cm^3^)	326.0 (186.8–547.5)	177.2 ± 76.1	562.5 ± 146.7	−4.7 ± 65.7
DXA-VAT mass (g)	301.5 (172.8–506.8)	163.9 ± 70.4	520.3 ± 136.6	−6.5 ± 63.3

Data are presented as mean ± SD or median (IQR). F, female; WC, waist circumference; WHR, waist to hip ratio; WHtR, waist to height ratio; MRI, magnetic resonance imaging; VAT, visceral adipose tissue; CSA, cross-sectional area; DXA, dual-energy X-ray absorptiometry. Body fat (%), android fat (kg), and android fat (%) were calculated using dual-energy X-ray absorptiometry. ^*∗*^*P* < 0.05, ^*∗∗*^*P* < 0.001 for pre-post intervention within group difference.

**Table 2:** Univariate and multiple linear regression illustrating the relationship between VAT CSA, volume, and area measured by MRI (gold standard) and DXA in all participants.

A	Variable	*r* value	*R* ^2^ value	Β (95% CI)	SE	*P* value
*MRI-VAT CSA*						
Model 1	DXA-VAT CSA	0.90	0.81	0.964 (0.840–1.087)	0.061	<0.001
Model 2						<0.001
	Body fat %	0.80	0.64	0.485 (0.119–0.851)	0.183	0.010
	DXA-VAT CSA	0.91	0.83	0.768 (0.579–0.957)	0.094	<0.001

*MRI-VAT volume*						
Model 3	DXA-VAT volume	0.90	0.82	0.979 (0.855–1.103)	0.062	<0.001
Model 4						<0.001
	Body fat %	0.82	0.68	0.636 (0.284–0.987)	0.175	0.001
	DXA-VAT volume	0.92	0.85	0.722 (0.540–0.904)	0.091	<0.001

*MRI-VAT mass*						
Model 5	DXA-VAT mass	0.90	0.81	0.979 (0.855–1.103)	0.062	<0.001
Model 6						<0.001
	Body fat %	0.82	0.68	0.637 (0.286–0.989)	0.175	0.001
	DXA-VAT mass	0.92	0.85	0.721 (0.540–0.903)	0.091	<0.001

SE, standard error; MRI, magnetic resonance imaging; DXA, dual-energy X-ray absorptiometry; VAT, visceral adipose tissue; CSA, cross-sectional area. Models 1, 3, and 5 describe linear regression results for baseline MRI-VAT where corresponding DXA-VAT outcomes were entered as dependent variables. Models 2, 4, and 6 describe multiple regression results for the aforementioned models, accounting for body fat %.

**Table 3:** Univariate and multiple linear regression illustrating the relationship between absolute and relative changes in VAT CSA, volume, and area measured by MRI (gold standard) and DXA in children with obesity following an intervention.

B	Variable	*r* value	*R* ^2^ value	B (95% CI)	SE	*P* value
*Absolute Δ MRI-VAT CSA*						
Model 1	Absolute Δ DXA-VAT CSA	0.27	0.07	0.302 (−0.148–0.752)	0.218	0.179
Model 2						0.140
	Absolute Δ body fat %	0.33	0.11	2.729 (−1.014–6.471)	1.813	0.145
	Absolute Δ DXA-VAT CSA	0.39	0.15	0.244 (−0.205–0.693)	0.218	0.272

*Relative Δ MRI-VAT CSA*						
Model 3	Relative Δ DXA-VAT CSA	0.36	0.13	0.569 (−0.030–1.168)	0.291	0.062
Model 4						0.041
	Relative Δ body fat %	0.39	0.15	1.910 (−0.314–4.134)	1.078	0.089
	Relative Δ DXA-VAT CSA	0.48	0.23	0.461 (−0.129–1.051)	0.286	0.120

*Absolute Δ MRI-VAT volume*						
Model 5	Absolute Δ DXA-VAT volume	0.26	0.07	0.254 (−0.137–0.646)	0.190	0.193
Model 6						0.141
	Absolute Δ body fat %	0.33	0.11	12.607 (−4.275–29.489)	8.180	0.136
	Absolute Δ DXA-VAT volume	0.39	0.15	0.199 (−0.188–0.587)	0.188	0.299

*Relative Δ MRI-VAT volume*						
Model 7	Relative Δ DXA-VAT volume	0.35	0.13	0.539 (−0.047–1.126)	0.285	0.070
Model 8						0.065
	Relative Δ body fat %	0.35	0.12	1.649 (−0.563–3.860)	1.072	0.137
	Relative Δ DXA-VAT volume	0.45	0.20	0.443 (−0.143–1.030)	0.284	0.132

*Absolute Δ MRI-VAT mass*						
Model 9	Absolute Δ DXA-VAT mass	0.25	0.07	0.234 (−0.132–0.600)	0.178	0.200
Model 10						0.150
	Absolute Δ body fat %	0.33	0.11	11.239 (−4.075–26.552)	7.420	0.143
	Absolute Δ DXA-VAT mass	0.38	0.15	0.176 (−0.189–0.541)	0.177	0.329

*Relative Δ MRI-VAT mass*						
Model 11	Relative Δ DXA-VAT mass	0.35	0.12	0.507 (−0.049–1.062)	0.270	0.072
Model 12						0.069
	Relative Δ body fat %	0.35	0.12	1.627 (−0.600–3.855)	1.079	0.145
	Relative Δ DXA-VAT mass	0.45	0.20	0.409 (−0.150–0.968)	0.271	0.144

SE, standard error; MRI, magnetic resonance imaging; DXA, dual-energy X-ray absorptiometry; VAT, visceral adipose tissue; CSA, cross-sectional area. Models 1, 5, and 9 describe linear regression results for absolute change in MRI-VAT where corresponding DXA-VAT outcomes were entered as dependent variables. Models 3, 7, and 11 describe linear regression results for relative change in MRI-VAT where corresponding DXA-VAT outcomes were entered as dependent variables. Models 2, 4, 6, 8, 10, and 12 describe multiple regression results for the aforementioned models, accounting for body fat %.

## Data Availability

The MRI and DXA data used to support the findings of this study are included within the article.
